# The First Confirmed Case of Coronavirus Disease 2019 (COVID-19) in an Adolescent with Abdominal Pain in Iran

**Published:** 2020-11

**Authors:** Shima Mahmoudi, Maryam Rostamyan, Alireza Aziz-Ahari, Babak Pourakbari, Mohammad Ghaffari, Setareh Mamishi

**Affiliations:** 1 Pediatric Infectious Disease Research Center, Tehran University of Medical Sciences, Tehran, Iran,; 2 Department of Infectious Diseases, Pediatrics Center of Excellence, Children's Medical Center, Tehran University of Medical Sciences, Tehran, Iran,; 3 Radiology Department, Rasool-e-Akram Hospital, Iran University of Medical Sciences, Tehran, Iran

**Keywords:** COVID-19, Children, Iran

## Abstract

**Background:**

The outbreak of coronavirus disease 2019 (COVID-19) is evolving rapidly. However, there is limited information about this disease in children and adolescents. Only a few pediatric cases of COVID-19 have been reported so far. Since the immune responses of children are different from adults, their clinical findings and therapeutic responses may differ. To the best of our knowledge, this is the first confirmed case of COVID-19 in a 12-year-old girl with abdominal pain in Iran.

**Case Presentation:**

A 12-year-old girl with a history of cold, dry cough, sore throat, fever, and left-sided abdominal pain was referred to the Children’s Medical Center, Tehran, Iran, on March 7, 2020. The chest X-ray indicated air space opacification in the right lower lobe and faint ground-glass opacity in the left lower lung. A subsequent chest computed tomography (CT) scan indicated blialteral patchy lower lobe consolidations. The patient’s oropharyngeal swab was positive for COVID-19, based on the result of real-time reverse transcription-polymerase chain reaction (rRT-PCR) assay. The patient’s clinical status was improved, and she was discharged five days after admission (March 11, 2020).

**Conclusion:**

Since the number of infected cases with COVID-19 is growing rapidly in Iran, early detection and management of infected cases are highly recommended for preventing the disease transmission and reducing the rate of infection.

## INTRODUCTION

Since December 2019, the outbreak of coronavirus disease 2019 (COVID-19), caused by a novel coronavirus, was reported in Wuhan, China and spread rapidly around the world. On March 11, the World Health Organization (WHO) declared this disease as a pandemic (1). In Iran, the epidemic of this disease was first reported on February 19, 2020. While the number of patients with COVID-19 infection is increasing, very few studies have examined pediatric cases (2).

According to statistics, the rate of infection is relatively low in children under the age of 18 years (2.4% of all cases) (3), and death seems to be rare in children (4). Although the prevalence of COVID-19 and its progression is low in children, they may be carriers of the virus to adults. Respiratory droplets and contact are the main transmission routes of severe acute respiratory syndrome coronavirus 2 (SARS-CoV-2); however, recent studies have indicated the possibility of fecal and oral transmission due to persistent shedding of SARS-CoV-2 in the stools of infected children (5).

There are several unanswered questions about the lower-than-expected prevalence of COVID-19 in children, its routes of transmission, and therapeutics. To the best of our knowledge, this is the first confirmed case of COVID-19 in a 12-year-old girl with abdominal pain in Iran.

## CASE SUMMARIES

A 12-year-old girl with a history of cold, dry cough, sore throat, fever, and left-sided abdominal pain (starting on March 4, 2020) was admitted to the Children’s Medical Center, an Iranian pediatric referral hospital in Tehran, Iran on March 7, 2020. She had no other underlying diseases before the onset of the disease. After admission to the hospital, fever and cough exacerbated, while no acute respiratory distress was observed (O_2_ saturation, 98%).

The patient’s laboratory findings were as follows: hemoglobin level: 13 g/dL; white blood cell (WBC) count: 5.6×10^3^/mm^3^; platelet count: 237,000/μL; partial thromboplastin time (PTT): 51 sec; prothrombin time (PT): 12.5 sec; international normalized ratio (INR): 1; C-reactive protein (CRP) level: 42 mg/L; erythrocyte sedimentation rate (ESR): 22 mm/h; lactate dehydrogenase (LDH): 456 IU/L; and creatine phosphokinase (CPK): 116 U/L. Also, lymphopenia (1400 cells/uL) was detected in the patient. The renal function and blood glucose were normal (blood urea nitrogen: 10 mg/dL; creatinine: 0.7 mg/dL; and blood sugar: 83 mg/dL).

**Figure 1.**
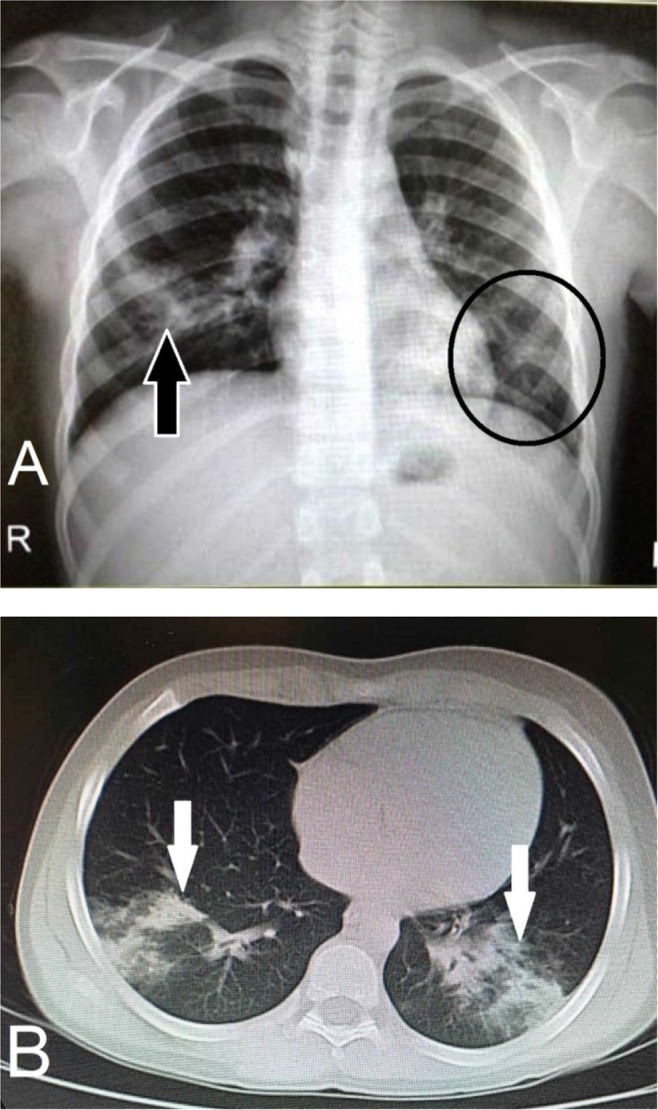
A) In chest X-ray radiography, air space opacification is visible in the right lower lobe (black arrow), and faint ground-glass opacity can be seen in the left lower lobe (encircled). B) The axial view of chest CT scan for the lungs shows patchy lower lobe consolidaions bilaterally (white arrows).

The chest X-ray revealed air space opacification in the right lower lobe, as well as faint ground glass opacity in the left lower lobe (Figure 1A). A subsequent chest computed tomography (CT) scan indicated patchy lower lobe consolidaions bilaterally (Figure 1B). An oropharyngeal swab was collected for real-time reverse transcriptase-polymerase chain reaction (rRT-PCR) assay of influenza and COVID-19. Saline infusion and empiric therapy with antibiotics, including amoxicillin/clavulanate (1 g loading everyday intravenously), azithromycin (200 mg), and beclomethasone nasal spray, were used initially for the patient.

On March 9, 2020, the patient's oropharyngeal swab was positive for COVID-19, based on the result of rRT-PCR assay. Accordingly, hydroxychloroquine (3 mg/kg) and oseltamivir (4 mg/kg) were administered twice per day for five days, as well as naproxen (250 mg every eight hours). The patient's clinical status improved, and she was discharged five days after admission to our hospital (March 11, 2020).

## DISCUSSION

The clinical characteristics of COVID-19 are not fully known, and children may have mild clinical manifestations after infection. Most adults and children with COVID-19 present with mild flu-like symptoms, and most cases without underlying diseases experience a mild disease (6). At the onset of disease, most cases have a good prognosis, and infected children mainly present with low to moderate fever (or no fever), fatigue, and cough, which may be followed by nasal congestion, runny nose, expectoration, diarrhea, and headache (7). However, abdominal pain has been rarely described (8).

Typically, normal or reduced WBC count, along with decreased lymphocyte count and normal or elevated CRP, may be among the laboratory findings of COVID-19 patients. Also, elevation of liver and muscle enzymes, as well as the elevated level of D-dimer, has been reported in severe cases (7). Chest imaging findings can help clinicians detect both early and severe stages of COVID-19, especially in areas with shortage of RT-PCR assays (9). Multiple small patchy shadows and interstitial changes may be observed in the early phase of the disease, while in severe cases, bilateral multiple ground-glass opacification, pulmonary infiltration shadows, and pulmonary consolidation with infrequent pleural effusion may be observed.

The main routes of COVID-19 transmission are infected patients, with or without clinical manifestations (7). The prevalence of positive COVID-19 cases is still growing in countries other than China, including Iran. Developing countries are facing major difficulties during this pandemic, and limited resources are a major problem that may lead to a high rate of healthcare-associated infections (10). Also, reducing the risk of nosocomial infection through preventive and control strategies is of critical importance.

The incidence of novel coronavirus pneumonia is higher in Asian populations than European and American populations, which may be explained by the higher expression level of angiotensin-converting enzyme 2 (ACE2) in Asians (9). It is difficult to determine the actual mortality rate of COVID-19, as it may be overestimated by examining only symptomatic cases; therefore, the number of infected cases may be higher than the reported statistics. Overall, the symptoms of COVID-19 emerge after an incubation period of roughly 5.2 days; however, this period is dependent on the age and immune system of the patient (2).

The most possible route of transmission for COVID-19 is person-to-person transmission by means of direct contact or spread of droplets by coughing or sneezing of an infected person; therefore, drastic measures are required to reduce the rate of person-to-person transmission to control the outbreak of COVID-19. Also, special attention must be paid to diminish the rate of transmission, especially in susceptible populations, including children, healthcare providers, and the elderly (2). Since patients infected with COVID-19 are the major source of disease transmission, children infected with this novel coronavirus must be quarantined at home or in separate rooms of designated clinics under the supervision of healthcare workers. Moreover, control of COVID-19 sources, hygiene, and sterilization are highly suggested.

## CONCLUSION

This is the first report of COVID-19 infection in an adolescent with abdominal pain in Iran. Since the number of infected cases is growing rapidly in Iran, early detection and management of infected patients are highly recommended for preventing transmission and reducing the rate of new infections.
